# Antidepressant effect of geranylgeranylacetone in a chronic mild stress model of depression and its possible mechanism

**DOI:** 10.3892/etm.2012.669

**Published:** 2012-08-16

**Authors:** JING-MEI ZHONG, SHAO-YUAN WU, JIE BAI, QIANG GUO, JIAN TAO, HUI CHEN, NAI-WEI ZHAO, ZHONG ZHAO, HAO FU

**Affiliations:** 1 Department of Neurology, The First People’s Hospital of Yunnan, Kunhua Affiliated Hospital of Kunming Medical University, Kunming 650032;; 2 Medical Faculty, Kunming University of Science and Technology, Kunming 650500, P.R. China

**Keywords:** depression, geranylgeranylacetone, heat shock protein 70, apoptosis, monoamine oxidase

## Abstract

Depression is a highly debilitating and widely distributed illness in the general population. Geranylgeranylacetone (GGA), a non-toxic anti-ulcer drug, has been reported to have protective effects in the central nervous system. The aim of this study was to determine the antidepressant effect of GGA in a chronic mild stress (CMS) model of depression. We confirmed that CMS in rats caused a reduction in locomotor activity and an increase in the levels of monoamine oxidase-A (MAO-A) and caspase-3 in the hippocampus. GGA treatment reversed stress-induced alterations in locomotor activity and target levels of MAO-A and caspase-3. In addition, GGA treatment induced heat shock protein 70 (Hsp70) expression in the hippocampus. These findings suggest that GGA possesses an antidepressant activity in a CMS model of depression. The activity of GGA in the relief of depression may be mediated via the induction of Hsp70 expression to suppress MAO-A expression and the apoptosis cascade.

## Introduction

Depression is a common mental disorder, with main characteristics including regular negative moods, decreased physical activity, loss of interest in usual activities, feeling of helplessness and suicidal tendencies (1). Although depression has been widely studied, the pathogenesis of depression remains unknown. Until now, antidepressants available on the pharmaceutical market mainly include tricyclic antidepressants, monoamine oxidase inhibitors, selective serotonin reuptake inhibitors and serotonin-noradrenergic reuptake inhibitors (2). However, numerous antidepressants frequently produce side-effects, including sedation, sleep disturbance, cognitive impairment and sexual dysfunction (3). Accordingly, the development of more effective antidepressants without any (or with fewer) adverse effects is required.

Heat shock protein 70 (Hsp70) functions as a molecular chaperone that mediates a highly conserved system of cellular responses to various stimuli (4). When the cell is exposed to stress, Hsp70 is induced to maintain cellular homeostasis (5,6). Several reports have shown that Hsp70 has a protective role in various models of nervous system injury, including oxidative stress and ischemic-reperfusion injury (6–8). In recent years, a huge body of evidence has accumulated suggesting that the activity of Hsp70 is associated with the pathogenesis of depression. The activity of the glucocorticoid receptor, which plays an important role in depression, is regulated by Hsp70 (9,10). The 162-base deletion in the 5′-flanking region of Hsp70 gene mRNA was observed in patients with depression (11). It also has been shown that antidepressants may increase Hsp70 expression, which is a possible mechanism underlying the therapeutic efficacy of antidepressants (12–14). As such, Hsp70 is a new therapeutic target for depression.

Geranylgeranylacetone (GGA) is an acyclic isoprenoid compound that has been widely used in clinic as an anti-ulcer drug. Numerous studies have demonstrated that GGA is a non-toxic Hsp70 inducer, which safely induces Hsp70 expression in gastric mucosa, intestine, liver, heart and retina (15–18). GGA is a lipid-soluble reagent and easily crosses the blood-brain barrier to exert neuroprotective activity. It has been reported that GGA administration induced the expression of heat shock proteins including Hsp70 and suppressed polyglutamine toxicity in cell culture and mouse models of spinal and bulbar muscular atrophy (19). GGA is also relevant to the treatment and prevention of various neural diseases, including ischemia-reperfusion injury and morphine addiction (20–22).

However, the effect of GGA on depression has not yet been investigated. In the present study, we suggest that when GGA is administered, it induces Hsp70, which may in turn alleviate behavioral abnormalities in depression. The present study was performed to investigate the possible antidepressant effects of GGA in the chronic mild stress (CMS) model of depression in rats.

## Materials and methods

### Reagents

GGA was purchased from Eisai (Tokyo, Japan). Antibodies [Hsp70, monoamine oxidase-A (MAO-A), caspase-3 and β-actin] were obtained from Santa Cruz Biotechnology, Inc. (Santa Cruz, CA, USA). DAB color developing reagent was purchased from Boao Seng Company (Beijing, China). TRIzol reagent was obtained from Molecular Research Center Company (Cincinnati, OH, USA). RevertAid™ First Strand cDNA Synthesis kit and dNTPS were purchased from Fermentas (Walldorf, Baden, Germany).

### Animals

Male Sprague-Dawley rats (Chengdu Dashuo Biological Technology Co., Ltd., Chengdu, China) weighing 180±20 g were used in the experiments. The rats were allowed to habituate to the housing facilities for 1 week before the experiments began. Rats were maintained on a 12 h light-dark cycle and had free access to food and water. The use of animals was performed in accordance with the National Institutes of Health Guide for the Care and Use of Laboratory Animals and approved by the Local Committee on Animal Use and Protection.

### CMS procedure

The method of stress was modified according to previous studies and previous practice in our laboratory. The stressed groups were subjected to the following stressors for three weeks: water and food deprivation for 24 h; physical restraint for 30 min (rats were placed into small iron compartments, which were 15-cm high and hand-made; the diameter of this compartment could be adjusted appropriately from 3–6 cm according to the size of rats); rotation for 30 min (motor-driven rotator, the velocity of rotation was 33 rpm); damp environment for 12 h (200 ml water was added to 100 g bedding); inclined cage for 12 h at 45°; the rats were combined with new invader rats for 24 h; daytime reversed and night reversed for 12 h, respectively. The order of each stress was randomly arranged and one type of stress was performed daily. The rats were randomly divided into 3 groups: the control group (control, n=12); the stress group (stress, n=12); the stress + 1,000 mg/kg GGA group (stress + GGA, n=12). The rats of the stress + 1,000 mg/kg GGA group were administered with GGA (1,000 mg/kg). The rats of the stress and control groups were injected intraperitoneally with saline (NS).

### Open field test

The open-field test was conducted in a four-sided 100×100×50 cm box, divided into 25 equilateral smaller squares. The test was conducted under a sedate environment. The rats were placed onto the center square of the box, then their activity was measured for 5 min. The number of crossings and rearings were scored manually. The apparatus was cleaned between tests.

### Immunohistochemistry analysis

The rats were sacrificed after the behavioral test by deep anesthesia. The brain samples were placed into the 4% paraformaldehyde for fixation overnight, followed by a wash with a flow of water and dehydration by gradient alcohol. The paraffin samples were sliced to a thickness of 5 μm. Endogenous peroxidase was blocked with blocking solution (3% H_2_O_2_ in methanol) for 15 min, following PBS washing. The slices were incubated in rabbit anti-mouse polyclonal Hsp70 (1:100 dilution) for 1 h at room temperature, and then incubated with biotinylated goat anti-rabbit IgG for 1 h. After rinsing with PBS, the slices were incubated with albumin fluid labeled with horseradish peroxidase for 1 h and then DAB solution was utilized for coloration. The slices were counterstained with hematoxylin.

### Reverse transcription-polymerase chain reaction (RT-PCR)

The rats were sacrificed after the behavioral test by deep anesthesia. The right hippocampus of rats was rapidly dissected out, frozen and stored in a deep freezer at −80°C until the assays. The total RNA of the hippocampus was isolated using TRIzol reagent following the manufacturer’s instructions. The RNA level was measured by an ultraviolet spectrophotometer by OD 260 measurements. cDNA was synthesized by reverse transcription. PCR amplification was performed with primers designed for the genes of interest. The primers used were as follows: HSP-70 sense, 5′-GCTGGTGAGCCACTTCGTG-3′ and antisense, 5′-TGGATCTGCGCCTTGTCC-3′ (Hsp70 PCR production, 288 bp); MAO-A sense, 5′-ATTGGAGGCGGCATC TCAGGAT-3′ and antisense, 5′-AGGTGGGAATGCACC ACGGAAT-3′ (MAO PCR production, 288 bp); caspase-3 sense, 5′-AACGAACGGACCTGTGG-3′ and antisense, 5′-TTT GCATGGAAA GTGGC-3′ (caspase-3 PCR production, 390 bp); β-actin sense, 5′-CACTGCCGCATCCTCTTCCTC-3′ and antisense, 5′-CTCCTGCTTGCTGATCCACAT-3′ (β-actin PCR production, 400 bp). The PCR products were separated by 2% agarose gel electrophoresis, visualized with ethidium bromide, and quantified using ImageJ software.

### Western blot analysis

The rats were sacrificed after the behavioral test by deep anesthesia. The right hippocampus of rats was rapidly dissected out, frozen and stored in a deep freezer at −80°C until the assays. Hippocampal tissue was homogenized in a solubilizing solution [20 mM Tris-HCl (pH 7.0), 25 mM β-glycerophosphate, 2 mM EGTA, 1% Triton X-100, 1 mM vanadate, 1% aprotinin, 1 mM phenylmethylsulfonyl fluoride, 2 mM dithiothreitol] on ice for 40 min. The lysate was centrifuged at 15,000 rpm for 15 min. The supernatant was denatured at 95°C for 5 min, then by 10 or 15% SDS-polyacrylamide gel electrophoresis and transferred to a PVDF membrance. Immunostaining was performed with anti-caspase-3 antibody, anti-MAO-A antibody, anti-Hsp70 antibody and β-actin antibody. Immunoreactivity was detected with peroxidase-conjugated secondary antibody in conjunction with chemiluminesence-based film autoradiography. For quantification, ImageJ software was used.

### Statistical analysis

All values are presented as means ± SD. Data were analyzed by ANOVA followed by a Tukey-Kramer test as the post hoc test. Differences were considered statistically significant at a level of P<0.05.

## Results

### Effects of GGA on the locomotor activity in the open-field test

**Figure 1:**
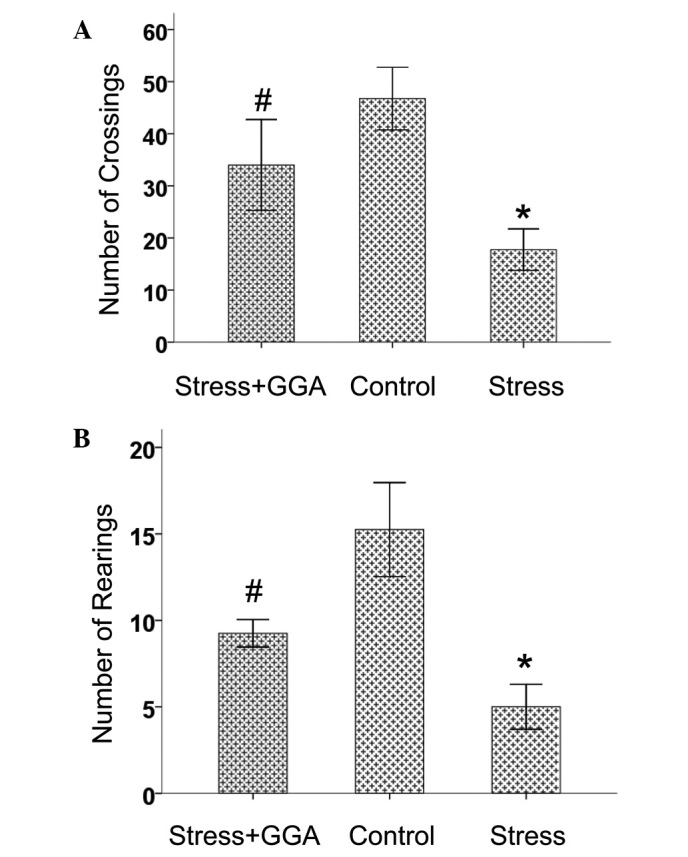
Effects of GGA on locomotor activity of rats after CMS. Animals were exposed to CMS and treated with GGA. (A) Number of crossings and (B) rearings were measured during the 5 min session. Values given are the means ± SD (n=12). ^*^P<0.05 as compared with the control; ^#^P<0.05 as compared with the stress group. CMS, chronic mild stress; GGA, geranylgeranylacetone.

Three weeks of CMS led to a significant decrease in locomotor activity compared with the control group, demonstrated by a decreased number of crossings and rearings (Fig. 1). By repeated treatment with GGA (1,000 mg/kg), changes in locomotor activity were almost completely reversed.

### Effects of GGA on MAO-A expression in the hippocampus

**Figure 2:**
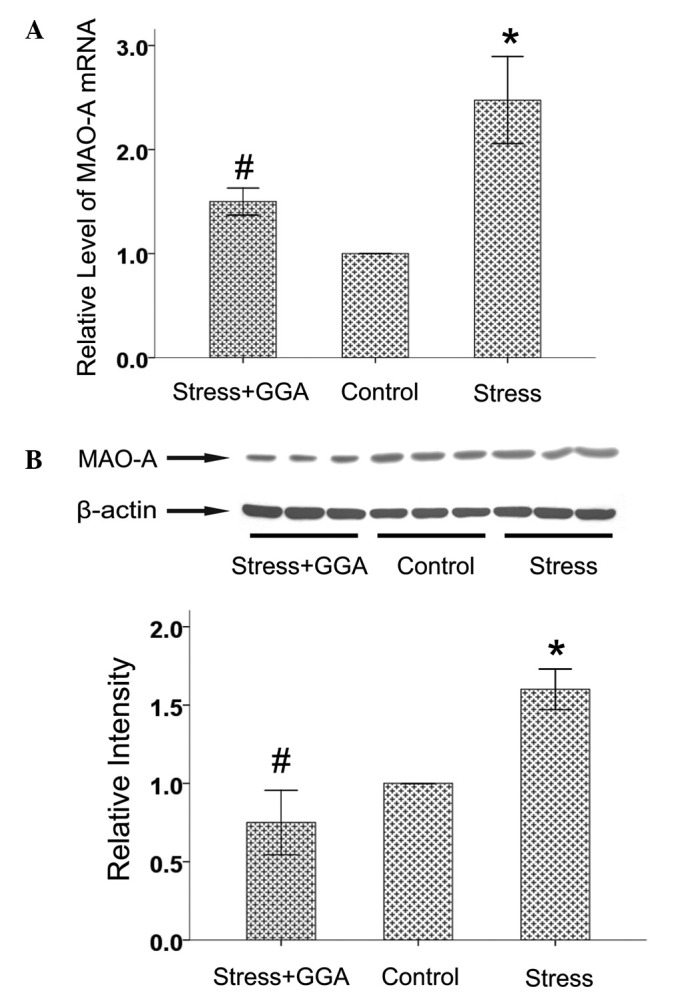
GGA suppressed CMS-induced increase in MAO-A level. After the open-field test, brains of rats (n=3) were dissected out. (A) The level of MAO-A mRNA in the hippocampus was detected by RT-PCR. (B) The level of MAO-A protein in the hippocampus was detected by western blot analysis, and quantification is shown in graphically. ^*^P<0.05 as compared with the control; ^#^P<0.05 as compared with the stress group. GGA, geranylgeranylacetone; CMS, chronic mild stress; MAO-A, monoamine oxidase-A.

It has been reported that there is an increased level of MAO-A in depression (23). RT-PCR and western blot analysis demonstrated that MAO-A mRNA and protein levels in the hippocampus were increased by CMS (Fig. 2). GGA treatment reversed these alterations, producing a significant decrease in MAO-A mRNA and protein levels in the hippocampus.

### Effects of GGA on caspase-3 expression in the hippocampus

**Figure 3:**
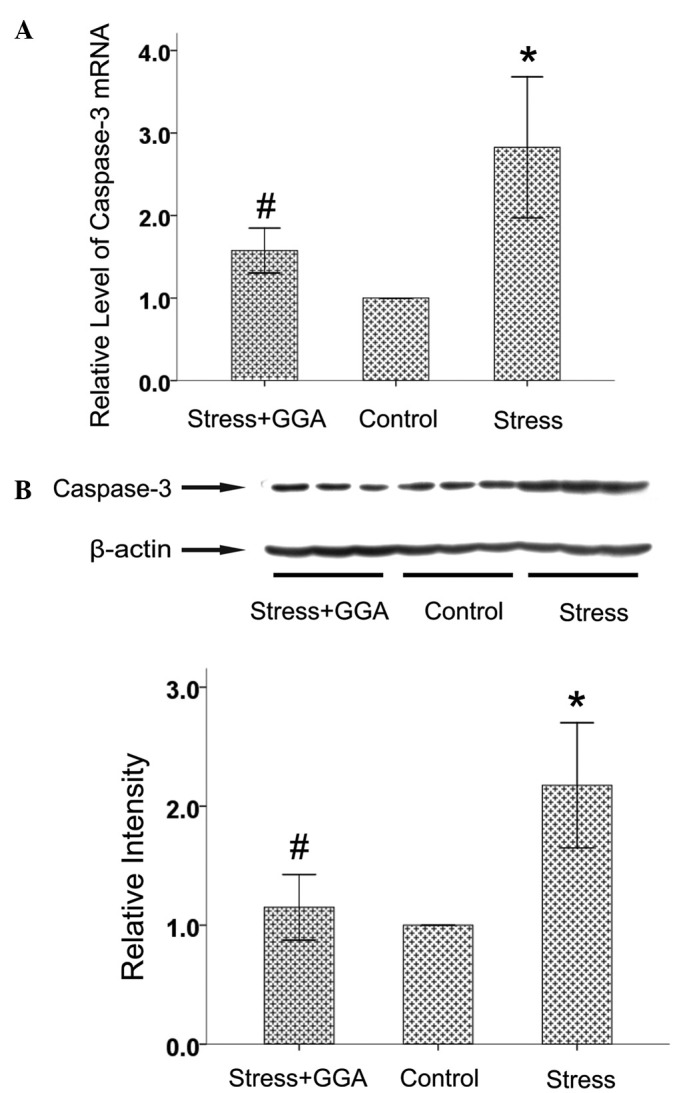
GGA suppressed CMS-induced increase in caspase-3 level. After the open-field test, brains of rats (n=3) were dissected out. (A) The level of caspase-3 mRNA in the hippocampus was detected by RT-PCR. (B) The level of caspase-3 protein in the hippocampus was detected by western blot analysis, and quantification is shown graphically. ^*^P<0.05 as compared with the control; ^#^P<0.05 as compared with the stress group. GGA, geranylgeranylacetone; CMS, chronic mild stress.

Neuronal apoptosis is involved in the pathogenesis of depression (24). Caspase-3 is a common downstream effector in the apoptosis cascade. Compared with the increased levels observed in the stress group, chronic GGA treatment resulted in a significantly decreased level of hippocampal caspase-3 mRNA and protein (Fig. 3). These results indicate that the antidepressant effect of GGA might be partly due to the suppression of neuronal apoptosis caused by CMS.

### Effects of GGA on Hsp70 expression in the hippocampus

**Figure 4:**
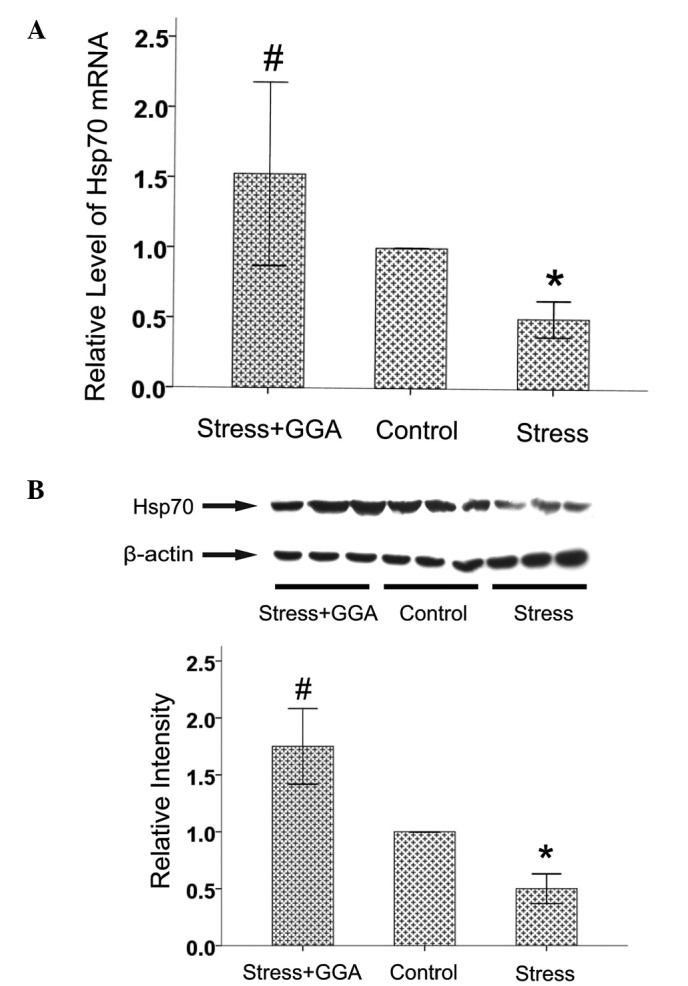
GGA increased Hsp70 expression. After the open-field test, brains of rats (n=3) were dissected out. (A) The level of Hsp70 mRNA in the hippocampus was detected by RT-PCR. (B) The level of Hsp70 protein in the hippocampus was detected by western blot analysis, and quantification is shown graphically. ^*^P<0.05 as compared with the control; ^#^P<0.05 as compared with the stress group. GGA, geranylgeranylacetone; Hsp70, heat shock protein 70.

**Figure 5:**
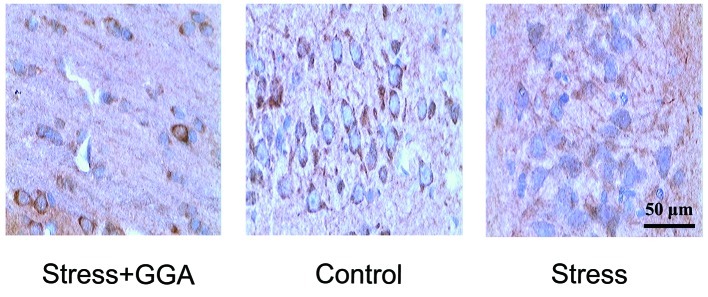
Representative photomicrographs of Hsp70 immunohistochemical staining in the hippocampus. After the open-field test, brains of rats (n=3) were dissected out. The level of Hsp70 protein in the hippocampus was detected by immunohistochemical analysis. Positive cells are represented as brown spots. Scale bar, 200 μm.

To investigate the role of Hsp70 in the antidepressant effect of GGA, we examined the Hsp70 level in the hippocampus after an open-field test by RT-PCR, western blot analysis and immunohistochemical analysis. GGA treatment significantly increased the levels of hippocampal Hsp70 mRNA and protein (Figs. 4 and 5). These data suggest that GGA induces Hsp70 expression, which may be involved in the antidepressant effect of GGA.

## Discussion

The current study demonstrates that GGA, an Hsp70 inducer, possesses an antidepressant effect. CMS in rats caused a reduction in locomotor activity and an increase in levels of monoamine oxidase-A and caspase-3 in hippocampus. GGA treatment (1,000 mg/kg) reversed stress-induced alteration in locomotor activity and levels of MAO-A and caspase-3. Previous animal studies hava reported that LD_50_ values of GGA is 15,000 mg/kg in oral dose and approximately 4,000 mg/kg in intraperitoneal injection in rats and mice (21). As such, although the dose used in the present study is high, it is not acutely toxic. Therefore, GGA may be a safe and potent agent for treating depression.

It has been widely reported that depression is a consequence of diminished neurotransmission due to a decrease in neurotransmitter concentrations (25,26). Among various neurotransmitters, monoamines (including noradrenaline, dopamine and serotonin) play crucial roles in the pathology of depression (27). Levels of monoamines are generally low in patients with depression (23). Monoamine oxidase (MAO), the most significant enzyme that catabolizes monoamines, is a trait-dependent indicator of vulnerability to depression (28). There are two forms of MAO: MAO-A and MAO-B. MAO-A activity is believed to be associated with depression, while MAO-B activity is believed to be associated with neurodegenerative diseases such as Parkinson’s disease (29,30). An elevated level of MAO-A is considered as the primary monoamine-lowering process in depression (23). In accordance with these reports, the present study observed that the levels of MAO-A mRNA and protein were increased in the hippocampus of CMS rats. In the present study, we also observed that GGA possessed an antidepressant effect and that the increased level of MAO-A caused by CMS was suppressed by GGA. These results suggest that the inhibitory effect of GGA on MAO-A expression may be a mechanism underlying antidepressant effects of GGA. However, further study is necessary to determine how GGA suppresses MAO-A expression. It has been reported that GGA increases thioredoxin-1, Hsp70 and prostaglandin expression in various cells, and exerts cytoprotection (15,20,31,32). In the present study, we observed that GGA induced Hsp70 expression in the hippocampus. However, further studies are required to investigate whether the inhibitory effect of GGA on MAO-A expression is due to the induction of Hsp70 and/or other inducing proteins.

Although the monoamine hypothesis provides a satisfactory explanation of the mechanism underlying the pathology of depression, alternative mechanisms are also involved in the pathology of depression. Recent reports have shown an association between depression, atrophy and cell loss in the brain (33–35). Loss of neuronal and glial density is observed in post-mortem brains of patients with depression (36–39). Chronic unpredictable stress leads to neuronal apoptosis in the cerebral cortex (40). The hippocampus is particularly sensitive to stress. It has been reported that there is a selective loss of hippocampal volume in depression (41,42). Loss of neurons is also observed in animal models of depression (41). It has been proposed that apoptosis is a contributing factor to the decrease in hippocampal volume and cell loss (43). Caspase-3 plays a crucial role in the execution of apoptosis (44). In the present study, we demonstrated that caspase-3 expression was increased in the hippocampus after CMS, suggesting the induction of apoptosis. Caspase-3 could be activated in the apoptotic cell by extrinsic (death ligand) and intrinsic (mitochondrial or endoplasmic reticulum) pathways (45–48). Further studies are required to determine which apoptosis pathway is involved in the CMS-induced apoptosis. A number of studies have shown that GGA protects against various stress injuries, including acoustic injury, ischemia, age-related hearing loss and 3-nitropropionic acid-induced cochlear damage, which involves Hsp70 induction (21,49,50). GGA is a lipid-soluble reagent that easily crosses the blood-brain barrier (50,51). In the present study, we observed that GGA had antidepressant effects. In addition, GGA induced Hsp70 expression in the hippocampus. The activation of caspase-3 caused by CMS was suppressed by GGA treatment. Hsp70 has been shown to inhibit the immediate apoptosis of cells exposed to numerous stresses in numerous tissues (52). The induction of Hsp70 expression by GGA may be associated with the inhibition of reactive oxygen species generation, toxicity of excitatory amino acid and apoptosis (8,53,54). Accordingly, the protective effect of GGA against the apoptosis cascade by inducing Hsp70 expression may be a mechanism underlying the antidepressant effects of GGA.

In conclusion, GGA exerted an antidepressant effect in the CMS model of depression in rats and this effect may be mediated by inducing Hsp70 expression to suppress MAO-A expression and the apoptosis cascade. In addition, these results suggest that attempts to develop Hsp70 inducers are beneficial for protection against depression.
